# Characterization of Chenopodin Isoforms from Quinoa Seeds and Assessment of Their Potential Anti-Inflammatory Activity in Caco-2 Cells

**DOI:** 10.3390/biom10050795

**Published:** 2020-05-21

**Authors:** Jessica Capraro, Stefano De Benedetti, Marina Di Dio, Elisa Bona, Ambra Abate, Paola Antonia Corsetto, Alessio Scarafoni

**Affiliations:** 1 Department of Food, Environmental and Nutritional Sciences, Università degli Studi di Milano, 20122 Milano, Italy; jessica.capraro@unimi.it (J.C.); stefano.debenedetti@unimi.it (S.D.B.); marinadidio93@gmail.com (M.D.D.); 2 Department of Sciences and Technology Innovation, Università del Piemonte Orientale, 13100 Vercelli, Italy; elisa.bona@uniupo.it; 3 Ambulatorio Polispecialistico Casazza, Casazza 24060, Italy; ambra.abate@postecert.it; 4 Department of Pharmacological and Biomolecular Sciences, Università degli Studi di Milano, 20122 Milano, Italy; paola.corsetto@unimi.it

**Keywords:** seed storage proteins, protein structure and function, food bioactives, *Chenopodium quinoa* Willd

## Abstract

Several food-derived molecules, including proteins and peptides, can show bioactivities toward the promotion of well-being and disease prevention in humans. There is still a lack of information about the potential effects on immune and inflammatory responses in mammalian cells following the ingestion of seed storage proteins. This study, for the first time, describes the potential immunomodulation capacity of chenopodin, the major protein component of quinoa seeds. After characterizing the molecular features of the purified protein, we were able to separate two different forms of chenopodin, indicated as LcC (Low charge Chenopodin, 30% of total chenopodin) and HcC (High charge Chenopodin, 70% of total chenopodin). The biological effects of LcC and HcC were investigated by measuring NF-κB activation and IL-8 expression studies in undifferentiated Caco-2 cells. Inflammation was elicited using IL-1β. The results indicate that LcC and HcC show potential anti-inflammatory activities in an intestinal cell model, and that the proteins can act differently, depending on their structural features. Furthermore, the molecular mechanisms of action and the structural/functional relationships of the protein at the basis of the observed bioactivity were investigated using in silico analyses and structural predictions.

## 1. Introduction

Quinoa (*Chenopodium quinoa* Willd.) seeds store about 12%–15% of proteins with high biological value [1,2]. The 11S globulins (about 37%–38% of the total proteins) and the 2S albumins (25%–31%) are the two major storage proteins in quinoa seed. The 11S globulin of quinoa seeds is also known by the trivial name of chenopodin, with an estimated native M*r* of about 320 kDa [1,3]. The 3D structure of chenopodin has not been yet determined. However, it likely assumes the typical hexameric quaternary structure of all 11S seed storage globulins, made of nearly identical subunits. Each subunit is composed of two polypeptides (designated as α and β chains) with M*r* around 30–40 and 20–23 kDa, respectively, linked by one disulfide bond [4,5]. The two chains of one subunit are encoded by a single gene, whose primary translation product (precursor) is later cleaved by a specific protease [6]. Like other seed storage proteins, chenopodin is codified by a family of genes, all expressed during seed development, and the native assembly of the holoprotein originates from the association of the resulting chains [5]. The hexameric structure arises from the assembly of two trimers formed by precursors only. The proteolytic processing of the precursors is the signal for two trimers to interact and form the hexamer [6].

Quinoa is an important domesticated crop of the Andean region, which recently has had a growing consumption in many countries [7,8]. The health benefits of quinoa seeds in human nutrition have been extensively reported [9], such that this crop is now indicated as a promising source for the development of functional foods and nutraceutical products [9,10,11]. Indeed, it is well-established that several food-derived molecules, including proteins and peptides, can show bioactivities toward the promotion of well-being and disease prevention in humans [12,13]. However, there is still a lack of information about the potential effects on immune and inflammatory responses in mammalian cells of proteins from quinoa seeds [12,14].

Inflammation is a protective nonspecific response of the immune system that can be triggered by a variety of factors, playing an important role in the body defense [15]. The nuclear factor κB (NF-κB) is a key agent in the beginning and maintenance of the inflammatory response in various tissues, including the intestinal mucosa [16,17]. NF-κB may target inflammation by inducing expression of inflammatory cytokines, chemokines, and adhesion molecules, and by regulating cell proliferation and differentiation [16,17]. Usually, inflammation resolves in a timely manner to avoid deleterious consequences [18]. Complete resolution of an acute inflammatory response and return to homeostasis are key processes in maintaining good health. Increased activation of NF-κB has often been detected as a hallmark of chronic inflammation, a condition ultimately leading to different inflammatory diseases [17,18]. Therefore, the downregulation of NF-κB activation may represent an attractive approach for anti-inflammatory therapies of the gut [19].

The present work pushes ahead with our line of research on the possible metabolic effects of food-derived molecules. In particular, this study aimed to investigate the potential capacity of chenopodin to modulate the NF-kb pathway in a human intestinal cell model following the trigger of inflammation. Furthermore, we speculated on the molecular mechanisms of action and on the structural/functional relationships of the protein at the basis of the observed bioactivity.

## 2. Materials and Methods

### 2.1. Chenopodin Extraction and Purification

The procedure was a modification of an unpublished protocol [20], in turn adapted from the original method for legume globulin extraction [21]. Quinoa seeds (var. Titicaca) were milled to a flour and sieved through a 60-mesh filter. All following procedures were performed at 4 °C. The flour was extracted twice with distilled water (1:20, *w*/*v*) for 4 h under stirring. The insoluble pellet obtained following centrifugation was then resuspended in the ratio 1:20 (*w*/*v*) in a 50 mM sodium phosphate buffer with pH of 7.5 containing 500 mM NaCl. The salt soluble globulins were extracted overnight under stirring. The suspension was centrifuged at 10,000 × *g* for 30 min. The ppt was discarded.

The supernatant was desalted using a Sephadex G-50 column, equilibrated in the 50 mM sodium phosphate buffer with pH of 7.5, and the proteins were separated by ion exchange chromatography (IEC) using a DEAE-cellulose column (20 × 180 mm) equilibrated with a 50 mM Tris-HCl buffer with pH of 8.0 (2 mg protein/mL resin). The protein elution was obtained using the same buffer with stepwise addition of 100 and 250 mM NaCl to avoid any chenopodin fractionation. Alternatively, chenopodin was fractionated adopting the same conditions but with stepwise addition of 100, 150, and 250 mM NaCl. The fractions containing the unretained proteins and that eluted with 100 mM NaCI were discarded. Those eluted with 150 and 250 mM NaCl were dialyzed against ammonium carbonate, freeze-dried, and kept in sealed tubes at 4 °C until used.

### 2.2. SDS-PAGE and IEF/SDS-PAGE

SDS-PAGE was carried out according to [22] on 12% polyacrylamide gel. Gels were stained with Coomassie Blue G-250 (Bio-Rad, Hercules, CA, USA) and the relative molecular masses of the polypeptides were determined by comparison with standard protein (GE Healthcare, Chicago, IL, USA): β-phosphorilase (94 kDa), BSA (66 kDa), egg albumin (45 kDa), carbonic anidrase (30 kDa), and trypsin inhibitor (20 kDa).

Bi-dimensional separations have been carried out according to [23]. Isoelectrofocalization (IEF) was performed on 11 cm, pH 3–10 linear Readystrips IPG strips (Bio-Rad), whereas the second dimension was performed on 12% polyacrylamide. Gels were stained with Coomassie Blue G-250.

### 2.3. Protein Identification

Bands were cut from the CBB-stained SDS-PAGE gel and destained overnight with a solution of 25 mM ammonium bicarbonate and 50% acetonitrile. The proteins were in-gel digested with trypsin (Roche, Segrate, Milano, Italy) as described in [24]. Mass spectrometry analyses were performed using a MicroLC 200 Plus Eksigent Technologies system (Sciex, Dublin, CA, USA) with a Halo Fused C18 column (0.5 × 100 mm, 2.7 μm). The LC system was interfaced with a 5600+ TripleTOF system equipped with a DuoSpray Ion Source (AB Sciex, Concord, Canada). Mass data were analyzed using Mascot server (Matrix Science Inc., Boston, MA, USA), available on-line at http://www.matrixscience.com/index.html, against an in-house sequence database prepared downloading all protein sequences present in NCBInr related to *Chenopodium quinoa*. Setting of possible variable modifications of proteins was as previously described [25].

### 2.4. Structural Predictions

Sequence analyses were performed with the online ProtParam tool [26] to determine isoelectric points and SignalP 5.0 to predict signal peptides [27].

Structural predictions were performed using the Swiss Model homology modelling pipeline [28], a tool available online via ExPASy server at https://swissmodel.expasy.org.

Electrostatic calculations were performed with APBS (Adaptive Poisson–Boltzmann Solver) [29] after structure preparation assigning atomic charges and radii with PDB2PQR [30]. Molecular surfaces were created with the MSMS package [31]. All these applications are part of the UCSF Chimera software (Version 1.13.1, The Regents of the University of California, Oakland, CA, USA), used for molecular graphics as well [32]. Docking analyses were performed with Patchdock and Firedock programs [33,34,35].

Sequence alignments were performed with T-Coffee Web Service through Jalview 2.11.1.0 [36,37], available at http://www.tcoffee.org and http://www.jalview.org/jalview-js/, respectively.

### 2.5. Caco-2 Cell Cultivation

Human intestinal epithelial Caco-2 cells were from the European Collection of Authenticated Cell Cultures (ECACC) (Merck, Milan, Italy). Cells were routinely grown in vented TC-treated 75 cm^2^ flasks (Star-lab, Milano, Italy) at 37 °C in a humidified atmosphere of 95% air and 5% CO_2_, using DMEM supplemented with 10% inactivated fetal bovine serum, 2mM L-glutamine, 100 U/mL penicillin, and 0.1 mg/mL streptomycin. All reagents were purchased from Sigma-Aldrich (Milan, Italy).

### 2.6. Transient Transfection and Immunomodulation Assay in Transfected Caco-2 Cells

The day before transfection, Caco-2 cells were seeded in 24-multiwell plates at a density of 2 × 10^5^ cells/cm^2^. Caco-2 cells were transiently transfected with the plasmid pNiFty2-Luc (InvivoGen, Rho, Italy). This plasmid combines five NF-κB binding sites with the luciferase reporter gene *luc*, thus the presence of NF-κB-activating molecules stimulates the expression of the luciferase gene. Transfection was performed using the StoS transfection kit (GeneSpin, Milan, Italy) following the manufacturer’s protocol. After transfection, cells were grown in complete medium as described before.

Immunomodulation assays were performed 24 h after transfection. Transfected Caco-2 cells were incubated with complete DMEM containing protein samples at a final concentration of 0.5 mg/mL and interleukin 1β (IL-1β) (10 ng/mL) for 4 h at 37 °C. After incubation, the plate was chilled on ice for 15 min and cells were scrapped mechanically from the bottom of wells, transferred into 1.5 mL tubes, and subjected to sonication for 10 s using a Soniprep 150 Ultrasonic Disintegrator (MSE) (Heathfield, East Sussex, UK). Insoluble particles were removed using centrifugation. One hundred μL of each supernatant were placed in a 96-well microtiter plate (PerkinElmer, Milano, Italy) and added with 12 μL of a 10 mM adenosine triphosphate (ATP) solution and 12 μL of 0.1 mM D-luciferin. The emitted bioluminescence was monitored every 120 s using a VICTOR3 1420 Multilabel Counter (PerkinElmer, Waltham, MA, USA). Each individual treatment was carried out in triplicate and each sample was analyzed in triplicate.

### 2.7. Gene Expression Studies by qPCR

Caco-2 cells were seeded in 12-multiwell plates at a density of 2 × 10^5^ cells/cm^2^ and allowed to reach confluence. Caco-2 cells were treated by adding quinoa proteins at a final concentration of 0.5 and 1.0 mg/mL, with or without IL-1β (20 ng/mL). Each individual treatment was carried out in triplicate and each sample was analyzed in triplicate.

After treatments, total RNA from Caco-2 cells was isolated using the Aurum Total RNA Kit (Bio-Rad). RNA was quantified at 260 nm using a fiber-optic ultra-micro cell (TrayCell, Hellma, Germany). One μg of total RNA was reverse transcribed to cDNA in 20 μL total volume using the iScript Reverse Transcription Supermix for RT-qPCR kit (Bio-Rad). Reaction conditions were: 25 °C for 5 min, followed by incubation at 46 °C for 20 min. Reactions were blocked at 95 °C for 1 min. cDNA samples were diluted 1:100 with sterile water before their use as templates for qPCR.

qPCR was carried out using a CFX Connect Real-Time PCR detection system (Bio-Rad, Hercules, CA, USA). Two µL of diluted cDNA samples were added to 10 μL of iQ SYBR Green Supermix (Bio-Rad). Each primer was added to a final concentration of 250 nM. Primers for amplification of IL-8 expressed gene were [38]: 5′-ATGACTTCCAAGCTGGCCGTGGCT-3′ and 5′-TCTCAGCCCTCTTCAAAAACTTCTC-3′. The GAPDH reference gene was amplified with the primer pair [39]: 5′-GGAAGGTGAAGGTCGGAGTC-3′ and 5′-CACAAGCTTCCCGTTCTCAG-3′. The reaction final volume was 20 μL. Cycling conditions were as follows: 3 min at 95 °C, then 40 cycles of denaturation (20 s at 95 °C), annealing (20 s at 55 °C), and extension (20 s at 72 °C).

Expression levels of the target genes in the test sample relative to the calibrator sample (untreated cells) were calculated according to the Livak method [40]. Each sample was analyzed in triplicate.

### 2.8. Determination of Saponins

Saponins were extracted according to [41] with minor modifications. Briefly, lyophilized protein samples were extracted with three chloroform/methanol mixtures (1:2, 2:1, and 1:1, *v*/*v*), dried, and partitioned with 1 vol of water and 2 vol of chloroform/methanol/water 3:48:47 (*v*/*v*/*v*). The organic phase was resuspended in chloroform/methanol 2:1 and then separated and detected using UPLC coupled to an ACQUITY QDa Mass Detector (Waters Corporation, Milford, MA, USA) on a BEH C18 column (Waters) according to [42].

### 2.9. Cell Vitality Assays

The assessment of Caco-2 cells vitality was determined as previously reported [43], by using both the (3-(4,5-dimethylthiazol-2-yl)-2,5-diphenyltetrazolium bromide (MTT) method and direct counts following staining with Trypan Blue.

### 2.10. Statistical Analysis

Data reported in the histograms are expressed as the means ± S.E. Data were analyzed by a *t*-test; *p* values < 0.05 were considered statistically significant. Data from RT-qPCR were analyzed using the CFX Maestro 1.1 software (Bio-Rad, Hercules, CA, USA).

## 3. Results

### 3.1. Characterization of the Isolate Chenopodin

**Figure 1:**
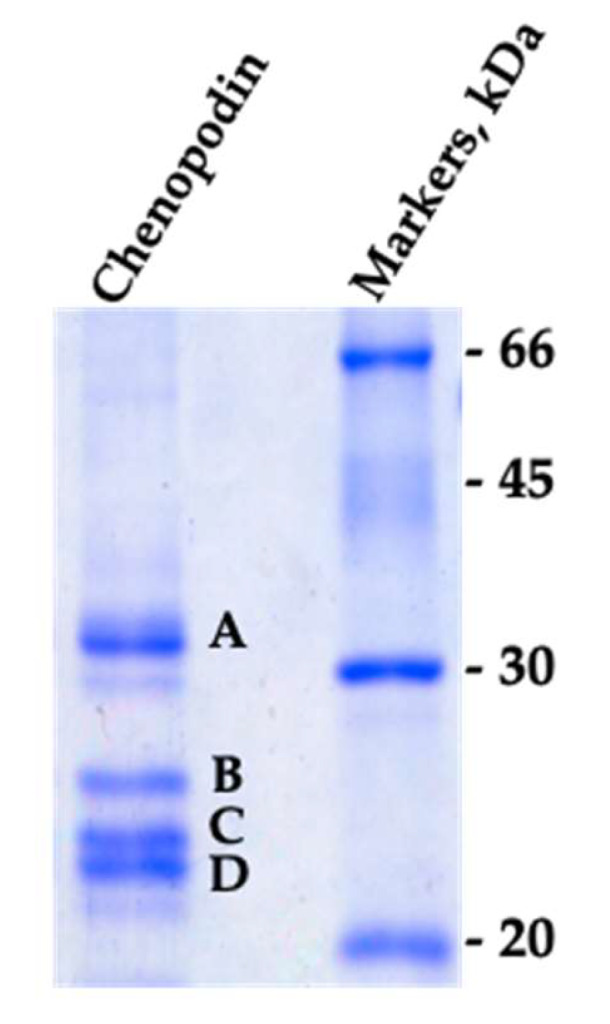
The SDS-PAGE showing the polypeptide composition of the purified chenopodin. The gel was run under reducing conditions. Letters indicate the bands analyzed by mass spectrometry for identification.

The electrophoretic pattern of chenopodin isolated by a single-step elution with 0.25 M NaCl (see Section 2.1) is shown in Figure 1. The result is in perfect accordance with previous data [5]. Two main groups of polypeptides with M*r* 35–37 (band A) and 22–25 kDa (bands B, C and D) are visible and correspond to the α and β chains of 11S seed globulin, respectively [3,5].

The four main bands (A, B, C and D) were excised from the gel and analyzed by mass spectrometry for protein identification. The results indicate that the bands all belong to the 11–13S globulin family (Table 1). More than one protein was present (up to four different gene products) in each of the four separated bands.

Chenopodin was further separated into two fractions, eluted from the IEC resin at distinct ionic strengths (0.15 and 0.25 M NaCl), thus differing for the net protein surface charge. M*r*s of the bands of these two fractions appear identical following SDS-PAGE (Supplementary Figure S1A), but differences concerning the isoelectric points (pIs) of the constituent polypeptides are clearly visible in IEF/SDS-PAGE bi-dimensional gels (Supplementary Figure S1B). From now on, the two fractions will be indicated as LcC (Low charge Chenopodin) and HcC (High charge Chenopodin). The relative amounts of LcC and HcC were about 30% and 70%, respectively. Starting from the mass spectrometry results, an attribution to LcC or HcC class was hypothesized based on the isoelectric point of the proteins identified, calculated with ProtParam tool from the sequence of the mature protein without the signal peptide, and predicted with SignalP-5.0 Server (Table 2).

Structures for the four chenopodin isoforms were generated by the SWISS-MODEL server, submitting the relative sequences with default parameters. For all structures, the model representing the homo-hexamer was chosen in order to fit the experimental data [3,5]. All the predicted models were obtained from the crystal structure of almond Pru1 protein (PDB: 3FZ3), which shares sequence identity ranging from 42.7% to 49.8%. Starting from the models, PDB2PQR software was used to calculate the proteins’ total charge at pH 8.0 to provide a further clue about the attribution of LcC and HcC classification. In the following modelling analyses, AAS67036.1 and XP_021752668.1 are assumed to represent LcC and HcC, respectively, in order to maximize and enhance the differences between the two classes.

**Figure 2:**
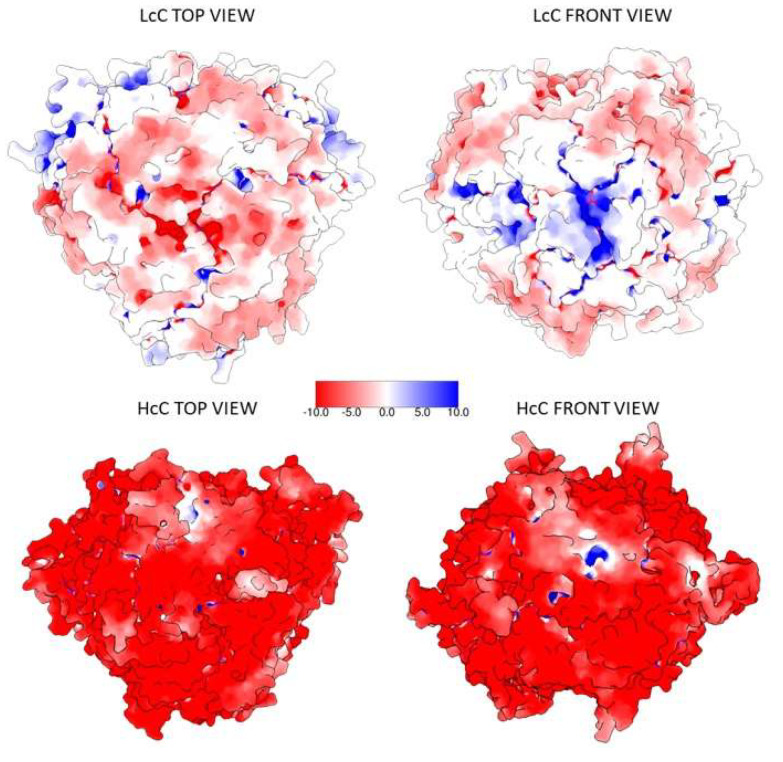
Electrostatic surface potential representation of the putative LcC (AAS67036.1) and HcC (XP_021752668.1) proteins, calculated at pH 8.0. Red: negative potential, blue: positive potential.

Proteins’ charges at pH 8.0 and atom radii were calculated with PDB2PQR in order to visualize the electrostatic surface potential with the APBS Electrostatic plugin implemented in the Chimera software. The results show that the negative charges of HcC are distributed all over the surface of the protein, allowing the interaction with the positively charged resin; conversely, LcC at pH 8.0 presents, in comparison, less solvent-accessible negatively charged residues (Figure 2; Table 2).

### 3.2. Immunomodulation Effects of Native Chenopodins

**Figure 3:**
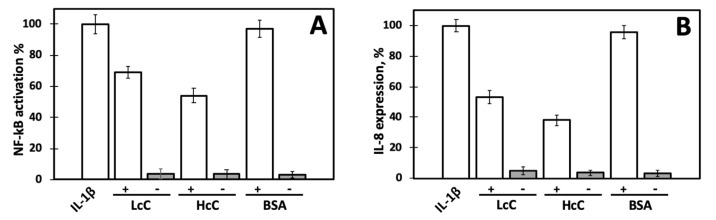
Inflammatory response of Caco-2 cells elicited by treatment with IL-1β alone and incubated with 0.5 mg/mL of LcC, HcC, or BSA with (+) or without (−) IL-1β. LcC or HcC and IL-1β were added to the cell growing medium at the same time. Response to IL-1β alone was set as 100%. See text for experimental details. (**A**) NF-kB activation percentage of transfected cells. (**B**) IL-8 expression percentage.

To study NF-κB activity, we transiently transfected Caco-2 cells with a NF-κB luciferase reporter construct. The pNiFty2-Luc plasmid includes five NF-κB binding sites, driving the expression of the luciferase reporter gene *luc*, thus the presence of NF-κB-activating molecules stimulates the expression of the luciferase gene. Following incubation with LcC and HcC proteins in the absence of IL-1β, NF-κB activation was found to be similar to that of the control cells (Figure 3A). The proteins themselves are not able to stimulate inflammation responses, and exert no cytotoxic effects, since insignificant losses of cell vitality were observed (Supplementary Figure S2). On the other hand, when the cells were stimulated by IL-1β, LcC and HcC were able to decrease NF-κB activation by about 30% and 45%, respectively. These data suggest that LcC and HcC are able to reduce NF-κB-mediated cellular inflammation with different capacities. As expected, bovine serum albumin (BSA), used as a control protein, did not exert any NF-κB modulating activity.

As a complementary approach, we also assessed the modulation of interleukin 8 (IL-8). The results are shown in Figure 3B and validate previous preliminary data [20]. IL-8 expression decreased to about 53% and 38% in the presence of LcC and HcC, respectively, confirming the capacity of chenopodin isoforms to protect Caco-2 cells from the inflammatory stimulus. No significant increase of IL-8 mRNA levels was detected in cells without stimulation by IL-1β, confirming that quinoa proteins do not exert pro-inflammatory effects on cells under the adopted experimental conditions. On the contrary, as expected, IL-8 expression markedly increased upon addition of IL-1β to the cell medium. A very similar result was obtained when the cells were treated with proteins at a final concentration of 1.0 mg/mL (Supplementary Figure S3).

The decreased activity of NF-κB caused by chenopodin induced the downregulation of IL-8 expression, being downstream from the NF-κB signaling pathway. IL-8 is a chemotactic cytokine produced by different cell lines, including intestinal epithelial and Caco-2 cells, following the NF-κB pathway activation [44,45]. Quinoa seeds contain high amount of saponins [46] that strongly interact with proteins [47] and have been associated with the inhibition of inflammatory mediator overproduction, including NO, TNF-α, and IL-6 [48]. We checked the presence of saponins in LcC and HcC samples, and they were not detected at the protein concentration used in the assays.

**Figure 4:**
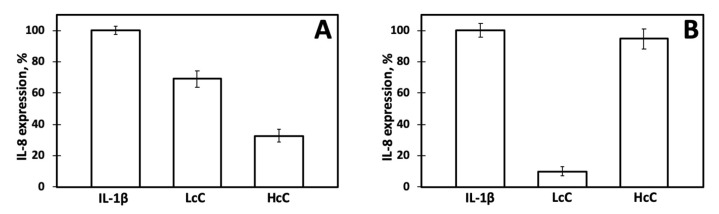
IL-8 relative expression in Caco-2 cells incubated with LcC or HcC (0.5 mg/mL) under different experimental conditions. Response to IL-1β alone was set as 100%. (**A**) LcC or HcC was added to the cell medium 30 min before the addition of IL-1β and then incubated for 1h. (**B**) LcC or HcC was first pre-incubated with IL-1β for 30 min and then added to the cell medium and incubated for 1h.

We then assessed the expression of IL-8 in Caco-2 cells incubated with IL-1β and quinoa proteins according to different experimental protocols. In the previous experiments (Figure 3B), either LcC or HcC and IL-1β were added to the cell growing medium at the same time. Otherwise, when chenopodins were added to the cell medium 30 min before the stimulation with IL-1β, expression of IL-8 was markedly decreased in cells treated with HcC, whereas the presence of LcC slightly affected IL-8 expression (Figure 4A). Finally, either LcC or HcC was first pre-incubated with IL-1β for 30 min and then added to the cell medium. In this latter condition, LcC showed higher anti-inflammatory effects with respect to HcC (Figure 4B).

### 3.3. In Silico Protein–Protein Interaction Analyses

**Figure 5:**
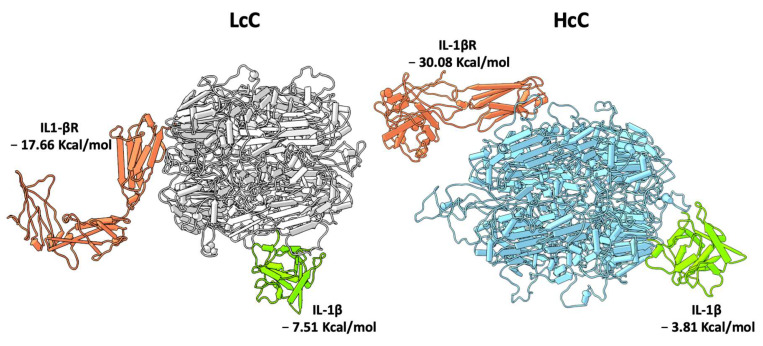
Molecular docking models. Pipes and plank representation of IL-1β (green) and the soluble IL-1β receptor (orange) binding to LcC (grey) and HcC (light blue). The binding energy for each complex is reported.

Molecular docking analyses were performed with the 3D models created for HcC and LcC to study their interaction with possible target molecules. IL-1β and the extracellular domain of IL-1β receptor (IL-1R, PDB: 1ITB) structures [49] were split in two different PDB files to obtain the single chain molecules for the interaction analyses with chenopodins. Both HcC and LcC are able to interact with either IL-1β or soluble IL-1R with different portions of the proteins. In greater detail, IL-1β binds to LcC with a more favorable binding energy (−7.51 Kcal/mol vs. −3.81 Kcal/mol), while conversely, binding of HcC to IL-1R is favored (−30.08 Kcal/mol vs. −17.66 Kcal/mol). Binding of the chenopodins to IL-1R is predicted to occur far from the binding site of IL-1β in a region close to the transmembrane domain, thus the potential mechanism of action could be exerted through the distortion of the receptor, rather than a competition with IL-1β for the binding site (Figure 5).

**Figure 6:**
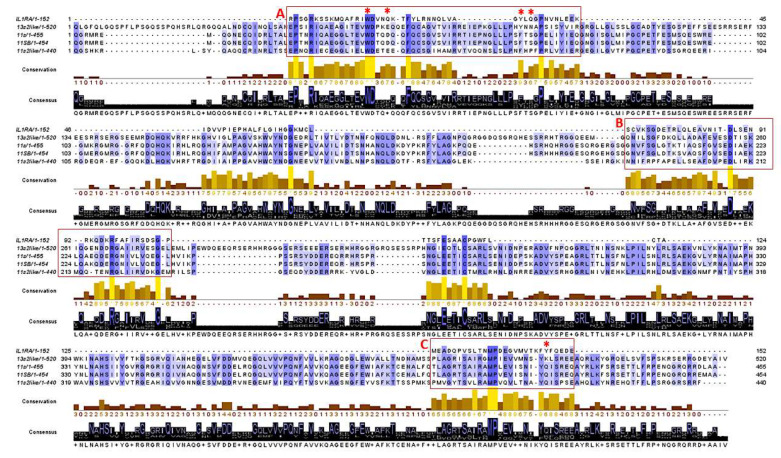
Alignment performed with the sequences of the mature form (without signal peptides) of IL-1RA (P18510) and the studied chenopodin isoforms. Sequence coloring represents percentage identity; conservation and consensus logos are shown. Regions with the highest homology are represented into boxes (A, B, and C), and amino acids crucial for IL1-RA interaction with IL-1R are marked with an asterisk. The acronyms of chenopodin sequences are those reported in Table 1.

The human interleukin-1 receptor antagonist (IL-1RA) is an antagonist of IL-1R [50], which acts by disrupting or preventing the formation of the complex between IL-1R and IL-1R accessory protein essential for the signal transduction [51]. Anakinra is a recombinant form of IL-1RA approved for the treatment of autoinflammatory disorders [52]. Its mechanism of action involves the competitive inhibition of the local inflammatory effects of IL-1β. In light of this, we compared the amino acid sequences of the four chenopodin isoforms and IL1-RA to investigate if chenopodins could mimic the agonist effect exerted by IL-1RA (Figure 6). Interestingly, three regions showed a high level of sequence similarity (Figure 6, boxes A, B, and C). These, although located quite far apart from one another within the sequences, are predicted to lie close to one another in the native conformation of chenopodin homo-hexamer (Supplementary Figure S4), suggesting a possible propensity of the region to be functional. In addition, five amino acids are crucial for the interaction of IL-1RA with IL-1R [53]. In chenopodins, all these amino acids are located in the regions with the highest similarities and four out of five are either conserved or substituted with similar ones (Figure 6).

**Figure 7:**
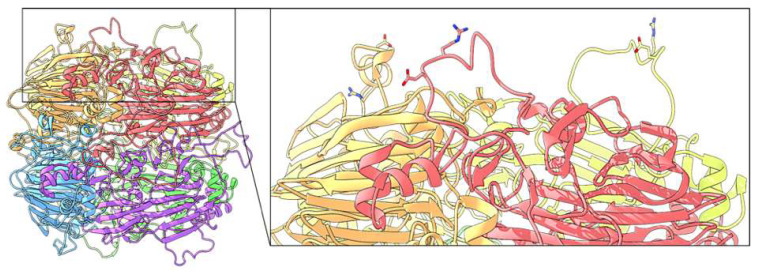
Predictive 3D structure of the homo-hexamer AAS67036 chenopodin. In the magnification panel on the right, a detail shows the surface locations of RGE sequences, one for each monomer, as stick representations.

Intriguingly, lunasin, a small water-soluble peptide of about 5000 Da initially isolated from soybeans but also identified in quinoa and other seeds, exerts anti-inflammatory activity on macrophage cells [54,55]. Lunasin can bind αVβ3 integrin through an Arg-Gly-Asp (RGD) motif [56]. This interaction has been associated with inhibition of inflammatory pathways involving NF-κB [56,57]. αVβ3 integrin is a receptor present in virtually all cells, including Caco-2 cells [58]. The amino acid sequence analysis of chenopodin isoforms investigated here indicated the presence of RGD in sequence XP_021752668.1. A similar motif Arg-Gly-Glu (RGE) is present in sequences AAS67036.1 and ABI94736.1. It has been demonstrated that RGE of lipid phosphate phosphohydrolase-3 is able to interact with both α5β1 and αVβ3 integrins, acting as a possible cell ligand site in humans and mice [59]. The 3D structural model of AAS67036.1 showed that RGE motif is predicted to be located in a very flexible and exposed loop at the protein surface (Figure 7).

XP_021752668.1 sequence analysis revealed the presence of an RGD motif in a non-homologous region at protein C-terminal that, despite being not modeled, could be, however, solvent-accessible.

## 4. Discussion

To date, few studies have reported on the anti-inflammatory properties of proteins and peptides from plant seeds. This study, for the first time, describes the potential immunomodulation capacity and anti-inflammatory effects of chenopodin, the major protein component of quinoa seeds. The results indicate that chenopodin may exert biological effects on intestinal cells and that the protein can act differently, depending on its structural features. Moreover, our results add new information about the structural characterization of chenopodin. The typical heterogenicity of seed storage proteins is clearly evident in chenopodin due to the multigenic origin and to post-translation proteolytic processing of precursor polypeptides [60]. Chenopodin is formed by a number of different polypeptides, as evidenced by electrophoretic analysis. We separated two main fractions, LcC and HcC, that differ for their net surface charge. All the constituent polypeptides are codified by at least four different genes. Structural modeling and electrostatic surface potential analysis support the possible attribution of the gene products to LcC and HcC.

Biological effects of LcC and HcC were investigated by measuring NF-κB activation and IL-8 expression studies in undifferentiated Caco-2 cells. This cell line, originally derived from colon carcinomatous enterocytes, has been exploited for a range of studies aimed to elucidate the molecular mechanisms of food-derived compounds, including the ability to elicit a reaction in response to pro-inflammatory stimuli [61]. Caco-2 cells express the IL-1R receptor [62,63]. In our case, inflammation was elicited using IL-1β, a pro-inflammatory cytokine able to strongly induce the release of IL-8 in Caco-2 cells by triggering the NF-κB signaling pathway [64]. Cytokine IL-1β is able to stimulate IL-8 release at both the mRNA and protein levels in Caco-2 cells [65]. Several reports have demonstrated the abilities of different natural compounds to regulate IL-8 expression by either transcriptional or post-transcriptional mechanisms in intestinal epithelium [14,61,66]. It was observed in IL-1β stimulated undifferentiated Caco-2 cells that the effects of olive oil phenols on IL-8 mRNA mirrored those observed on IL-8 release [61].

Overall, the results outline possible mechanisms through which LcC and HcC may modulate inflammation response. In our experimental conditions, LcC likely binds to IL-1β, and prevents cytokine interaction with the target membrane receptor. HcC seems to instead interact directly with the cells, possibly reducing the accessibility to IL-1β. The carried out predictive molecular modelling supports this possible mechanism of action.

Saponins in LcC and HcC samples were not detected. Thus, the protein purification process effectively removed saponins and, therefore, the observed biological activities are attributable only to the proteins.

Although our results suggest the activation of the canonical NF-κB signaling pathway, the involvement of the non-canonical pathway [67] cannot be excluded. However, several studies showed that some food proteins may stimulate members of the Toll-like receptor family (TLRs) such as pea proteins and peptides obtained from milk [68]. It has been shown that the inhibitory effect on IL-8 cell expression can be due to a direct interaction of whey protein hydrolysates with TLR4 [69]. Plant extracts were able to modulate TLR signaling as well through the direct activation of receptors or further downstream of TLRs’ pathways [70]. On the other hand, though, other events or factors cannot be ruled out. One possibility is that LcC and HcC may be internalized in the cytoplasm, as it occurs for other seed proteins [71], exerting their activity in intracellular pathways such as in IkB translocation [72], for example.

Since the evaluation of the long-term effects of chenopodins on the inflammatory response was out of the scope of the work, protein accumulation of inflammatory effectors such as IL-8, IL-6 and TNF-α has not been investigated.

Whether or not chenopodin can actually exert biological effects on the human body following ingestion remains to be established. Conformational changes or denaturations induced by food processing and gastrointestinal digestion may have important consequences on the observed activity that need to be deeply investigated. Digestibility of isolated quinoa proteins is, indeed, higher if compared to those of whole meal quinoa flour [73]. Further studies have been undertaken to shed light on the molecular mechanisms of action and the long-term effects of chenopodin on inflammatory response using different intestinal cell models and assessing a panel of inflammation protein markers. This assessment is envisaged because the correspondence between transcript and protein levels is not always maintained. A little variation in transcription levels, indeed, could have remarkable effects on protein accumulation and, in the end, on the cellular events involved in prolonged inflammation.

## Figures and Tables

**Table 1:** List of proteins identified by mass spectrometry (MS). More than one protein was found in each of the four (**A**–**D**) separated bands. MWs refers to the unprocessed precursors as reported by data banks. Statistical information, experimental data, and sequences of the MS attributions are provided in Supplementary Table S1.

Band	Accession ID	Description	MW
**A**	AAS67036.1	11S seed storage globulin	54007
	ABI94736.1	11S seed storage globulin B	53942
	XP_021768838.1	11S globulin seed storage protein 2-like	52806
	XP_021752668.1	13S globulin seed storage protein 2-like	61191
**B**	XP_021752668.1	13S globulin seed storage protein 2-like	61191
**C**	AAS67036.1	11S seed storage globulin	54007
	ABI94736.1	11S seed storage globulin B	53942
	XP_021768838.1	11S globulin seed storage protein 2-like	52806
	XP_021752668.1	13S globulin seed storage protein 2-like	61191
**D**	AAS67036.1	11S seed storage globulin	54007
	ABI94736.1	11S seed storage globulin B	53942

**Table 2:** Isoelectric point and charge calculated at pH 8.0 of the identified chenopodin isoforms; *e* is the charge of one electron.

Accession ID	Description	pI	Charge
XP_021752668.1	13S globulin seed storage protein 2-like	5.96	−101 *e*
XP_021768838.1	11S globulin seed storage protein 2-like	6.35	−79 *e*
ABI94736.1	11S seed storage globulin B	6.47	−45 *e*
AAS67036.1	11S seed storage globulin	7.34	−38 *e*

## Supplementary Materials

The following are available online at https://www.mdpi.com/2218-273X/10/5/795/s1, Figure S1: Mono-dimensional and bi-dimensional electrophoresis of LcC and HcC chenopodin fractions, Figure S2: Assessment of Caco-2 cell vitality following treatment with LcC and HcC, Figure S3: Relative expression of Il-8 in Caco-2 cells incubated with 1.0 mg/mL LcC or HcC, Figure S4: Magnification of the predictive 3D structure of the homo-hexamer AAS67036 chenopodin (HcC) showing the regions with the highest homology with IL-1RA, Table S1: List of the proteins identified by MS.
