# Supplementary material for: Characterization of Chenopodin Isoforms from Quinoa Seeds and Assessment of Their Potential Anti-Inflammatory Activity in Caco-2 Cells
Source: Biomolecules. 2020 May 21;10(5):795. doi: 10.3390/biom10050795 (PMC7277664; doi:10.3390/biom10050795)

Supplementary Figure S1: Mono-dimensional (A)and bi-dimensional (B) electrophoretic separations of LcC an HcC chenopodin fractions

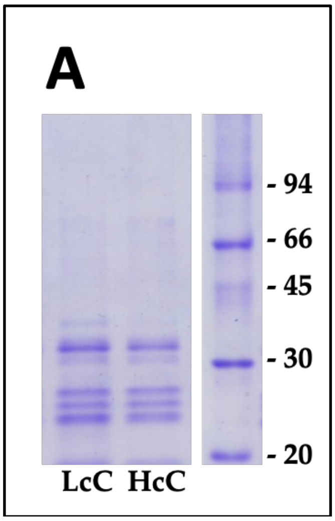

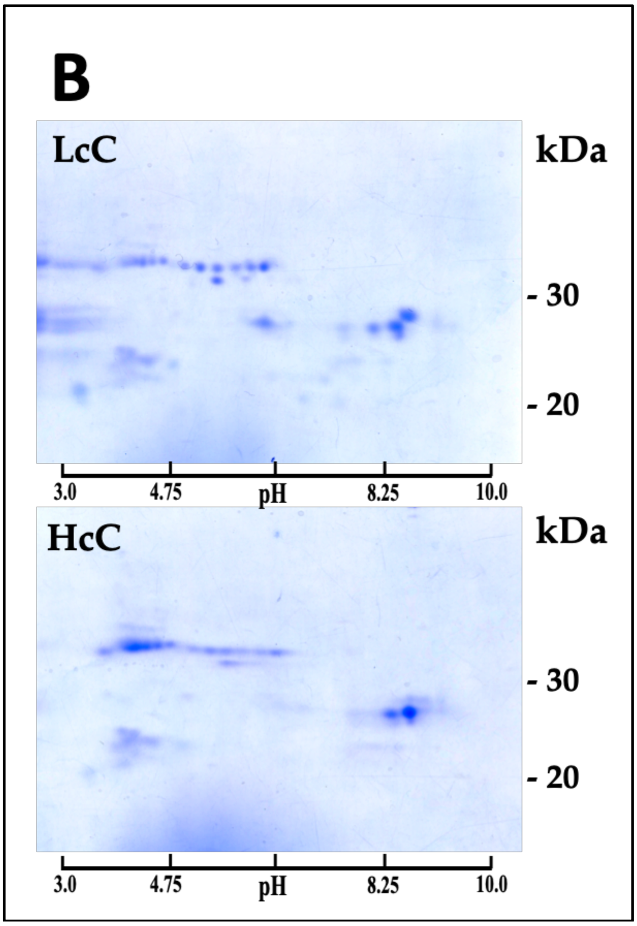

Supplement: Supplementary file 1 [file biomolecules-10-00795-s001.zip › Supplementary Figure S1.pdf]
