# Supplementary material for: Characterization of Chenopodin Isoforms from Quinoa Seeds and Assessment of Their Potential Anti-Inflammatory Activity in Caco-2 Cells
Source: Biomolecules. 2020 May 21;10(5):795. doi: 10.3390/biom10050795 (PMC7277664; doi:10.3390/biom10050795)

Supplementary Figure S2. Assessment of Caco-2 cell vitality following treatment with LcC and HcC. 3-[4–dimethylthiazol-2-yl]-2,5-diphenyltetrazolium bromide (MTT) method (white bars) and cell counting upon staining with Tryphan Blue (gray bars) have been used. LcC and HcC were both used at 0.5 mg/mL and 1.0 mg/mL (final concentration). Caco-2 cells used as the control were incubated without protein addition (0 mg/mL). Determinations have been carried out in triplicate according to ref. 43. Data are expressed as a percentage of the control sample.

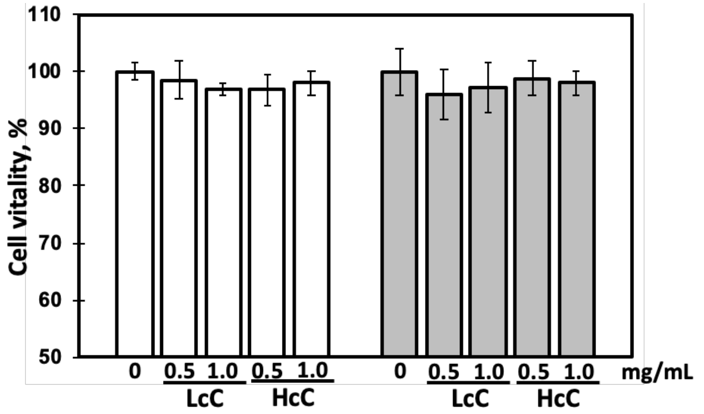

Supplement: Supplementary file 1 [file biomolecules-10-00795-s001.zip › Supplementary Figure S2.pdf]
