# Supplementary material for: Characterization of Chenopodin Isoforms from Quinoa Seeds and Assessment of Their Potential Anti-Inflammatory Activity in Caco-2 Cells
Source: Biomolecules. 2020 May 21;10(5):795. doi: 10.3390/biom10050795 (PMC7277664; doi:10.3390/biom10050795)

Supplementary Figure S3. IL-8 relative expression in Caco-2 cells incubated with LcC or HcC (1.0 mg/mL) under different experimental conditions. Response to IL-1β alone was set as 100%.

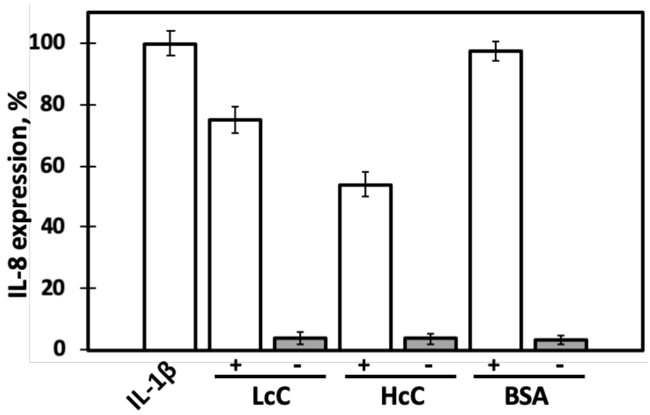

Supplement: Supplementary file 1 [file biomolecules-10-00795-s001.zip › Supplementary Figure S3.pdf]
