# Supplementary material for: Characterization of Chenopodin Isoforms from Quinoa Seeds and Assessment of Their Potential Anti-Inflammatory Activity in Caco-2 Cells
Source: Biomolecules. 2020 May 21;10(5):795. doi: 10.3390/biom10050795 (PMC7277664; doi:10.3390/biom10050795)
Supplement: Supplementary file 1 [file biomolecules-10-00795-s001.zip › Supplementary Figure S4.pdf]

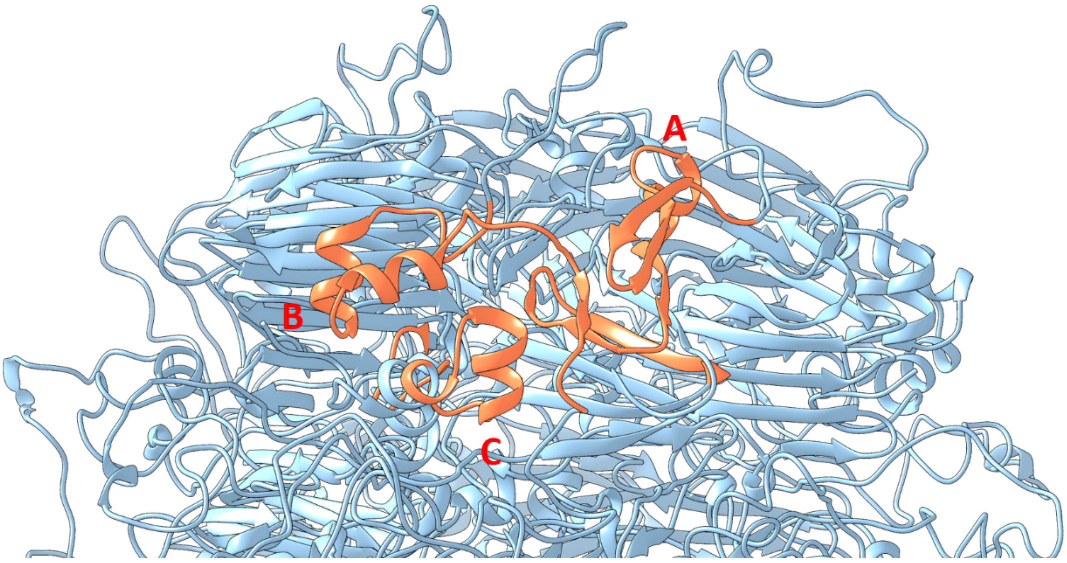

Supplementary Figure S4. Magnification of the predictive 3D structure of the homo-hexamer AAS67036 chenopodin (HcC), showing the regions with the highest homology with IL-1RA. Regions A, B and C are represented in coral, to show their spatial closeness. Sequences A and B belong to one monomer, whereas sequence C are part of an adjacent monomer.
